# Predicting EGFR Status After Radical Nephrectomy or Partial Nephrectomy for Renal Cell Carcinoma on CT Using a Self-attention-based Model: Variable Vision Transformer (vViT)

**DOI:** 10.1007/s10278-024-01180-0

**Published:** 2024-06-28

**Authors:** Takuma Usuzaki, Ryusei Inamori, Mami Ishikuro, Taku Obara, Eichi Takaya, Noriyasu Homma, Kei Takase

**Affiliations:** 1 Department of Diagnostic Radiology, Tohoku University Hospital, Sendai, Japan; 2 Department of Clinical Imaging, Graduate School of Medicine, Tohoku University, Sendai, Miyagi Japan; 3 Division of Molecular Epidemiology, Graduate School of Medicine, Tohoku University, Sendai, Miyagi Japan; 4 Division of Molecular Epidemiology, Department of Preventive Medicine and Epidemiology, Tohoku Medical Megabank Organization, Tohoku University, Sendai, Japan; 5 Department of Pharmaceutical Sciences, Tohoku University Hospital, Sendai, Japan; 6 AI Lab, Tohoku University Hospital, Sendai, Japan; 7 Tohoku University Hospital, 1-1 Seiryo-Machi, Aoba-Ku, Sendai, Miyagi 980-8574 Japan

**Keywords:** Renal cell carcinoma (RCC), Deep learning, Machine learning, Vision transformer, Estimated glomerular filtration rate (eGFR)

## Abstract

**Objective:**

To assess the effectiveness of the vViT model for predicting postoperative renal function decline by leveraging clinical data, medical images, and image-derived features; and to identify the most dominant factor influencing this prediction.

**Materials and Methods:**

We developed two models, eGFR10 and eGFR20, to identify patients with a postoperative reduction in eGFR of more than 10 and more than 20, respectively, among renal cell carcinoma patients. The eGFR10 model was trained on 75 patients and tested on 27, while the eGFR20 model was trained on 77 patients and tested on 24. The vViT model inputs included class token, patient characteristics (age, sex, BMI), comorbidities (peripheral vascular disease, diabetes, liver disease), habits (smoking, alcohol), surgical details (ischemia time, blood loss, type and procedure of surgery, approach, operative time), radiomics, and tumor and kidney imaging. We used permutation feature importance to evaluate each sector's contribution. The performance of vViT was compared with CNN models, including VGG16, ResNet50, and DenseNet121, using McNemar and DeLong tests.

**Results:**

The eGFR10 model achieved an accuracy of 0.741 and an AUC-ROC of 0.692, while the eGFR20 model attained an accuracy of 0.792 and an AUC-ROC of 0.812. The surgical and radiomics sectors were the most influential in both models. The vViT had higher accuracy and AUC-ROC than VGG16 and ResNet50, and higher AUC-ROC than DenseNet121 (p < 0.05). Specifically, the vViT did not have a statistically different AUC-ROC compared to VGG16 (p = 1.0) and ResNet50 (p = 0.7) but had a statistically different AUC-ROC compared to DenseNet121 (p = 0.87) for the eGFR10 model. For the eGFR20 model, the vViT did not have a statistically different AUC-ROC compared to VGG16 (p = 0.72), ResNet50 (p = 0.88), and DenseNet121 (p = 0.64).

**Conclusion:**

The vViT model, a transformer-based approach for multimodal data, shows promise for preoperative CT-based prediction of eGFR status in patients with renal cell carcinoma.

**Supplementary Information:**

The online version contains supplementary material available at 10.1007/s10278-024-01180-0.

## Introduction

Deterioration in renal function is one of the most common complications following partial nephrectomy (PN) or radical nephrectomy (RN). Preoperative estimation of postoperative renal function plays an important role in decision-making for patients with renal tumors [1–3]. This estimation provides clinicians with important insights when evaluating whether surgical treatment is beneficial for a patient [4]. Multiple prediction models have been proposed to estimate the impact of surgical treatment on renal function. Shimada et al. proposed a formula to accurately predict eGFR at 1 year after RN in each sex using a stepwise regression model [5]. They identified preoperative eGFR and age as significant predictors of deteriorating renal function after RN in both males and females, whereas tumor size and body mass index (BMI) were significant predictors only in males [5]. These results indicate that prediction of postoperative eGFR should include take into account various patient characteristics in addition to image-derived features such as tumor size. Deep learning is another promising technique for estimating postoperative eGFR. Kuo et al. proposed a deep learning approach for automatically determining estimated glomerular filtration rate (eGFR) and chronic kidney disease (CKD) status using ultrasound images. The overall classification accuracy for CKD status using their proposed model was 85.6%, which is higher than that of experienced nephrologists (60.3%–80.1%) [6]. These results suggest that medical images contain information relevant to eGFR, but there is limited research regarding which clinical information, medical images, and image-derived features can be simultaneously analyzed to predict deterioration in renal function. When only the patient characteristics are used as input, the image-derived features are not considered. Conversely, when the input relies solely on images, important patient characteristics are not incorporated in the prediction of renal impairment. Therefore, an accurate preoperative estimation of postoperative renal function remains an unmet clinical need.

To overcome these challenges, and inspired by ViT, variable Vision Transformer (vViT) was proposed as a model capable of analyzing multiple sequences of different dimensions [7–9, 22, 23]. Originally introduced for natural language tasks, transformer architectures have been shown to be ideally suited for combining imaging and non-imaging data [10]. These architectures feature a self-attention mechanism to model long-range dependencies, which is better than conventional models at understanding characteristic features of multimodal data such as contextual information and medical images.

The vViT can separately analyze input factors such as clinical information, medical images, and image-derived features. Combining vViT with feature importance analysis enables determination of the dominant factor among numerous input factors; i.e., for identifying the dominant factor in predicting deterioration in renal function among clinical information, medical images, and image-derived features.

The aim of this study is to investigate the performance of vViT in predicting deterioration in postoperative renal function by utilizing clinical information, medical images, and image-derived features. Additionally, the study seeks to identify the most dominant factor contributing to prediction of deterioration in postoperative renal function.

## Material and Methods

### Data collection and Selection Criteria

#### Data Collection

All data used in this cross-sectional study were obtained from the training set of the 2019 Kidney and Kidney Tumor Segmentation Challenge (C4KC-KiTS), following The Cancer Image Archive data usage policy and restrictions [11]. We included all available 210 patients with demographics, clinical data, and contrast-enhanced CT (Computed Tomography) images from the datasets, the details of which are described elsewhere [11]. The retrospective data collections were approved by the institutional review board of the University of Minnesota [11].

#### Selection Criteria

The following exclusion criteria were applied in construction of the model: (i) incomplete data for any of characteristics (age, sex, and BMI), disease history (peripheral vascular disease history, diabetes mellitus history, and liver disease history), habit information (smoking history, and alcohol intake), and surgical information (ischemia time, estimated blood loss, surgical type, surgical procedure, surgical approach, and operative time); (ii) incomplete data for any preoperative or postoperative eGFR; (iii) radiomic features could not be extracted from the arterial phase contrast-enhanced CT images and annotation image of a tumor contained fewer than 256 pixels; and (iv) number of images beyond the mean ± standard deviation. In addition to these criteria, random selection (v) was performed to equalize the number of images classified in each category.

### Model Architecture

**Fig. 1:**
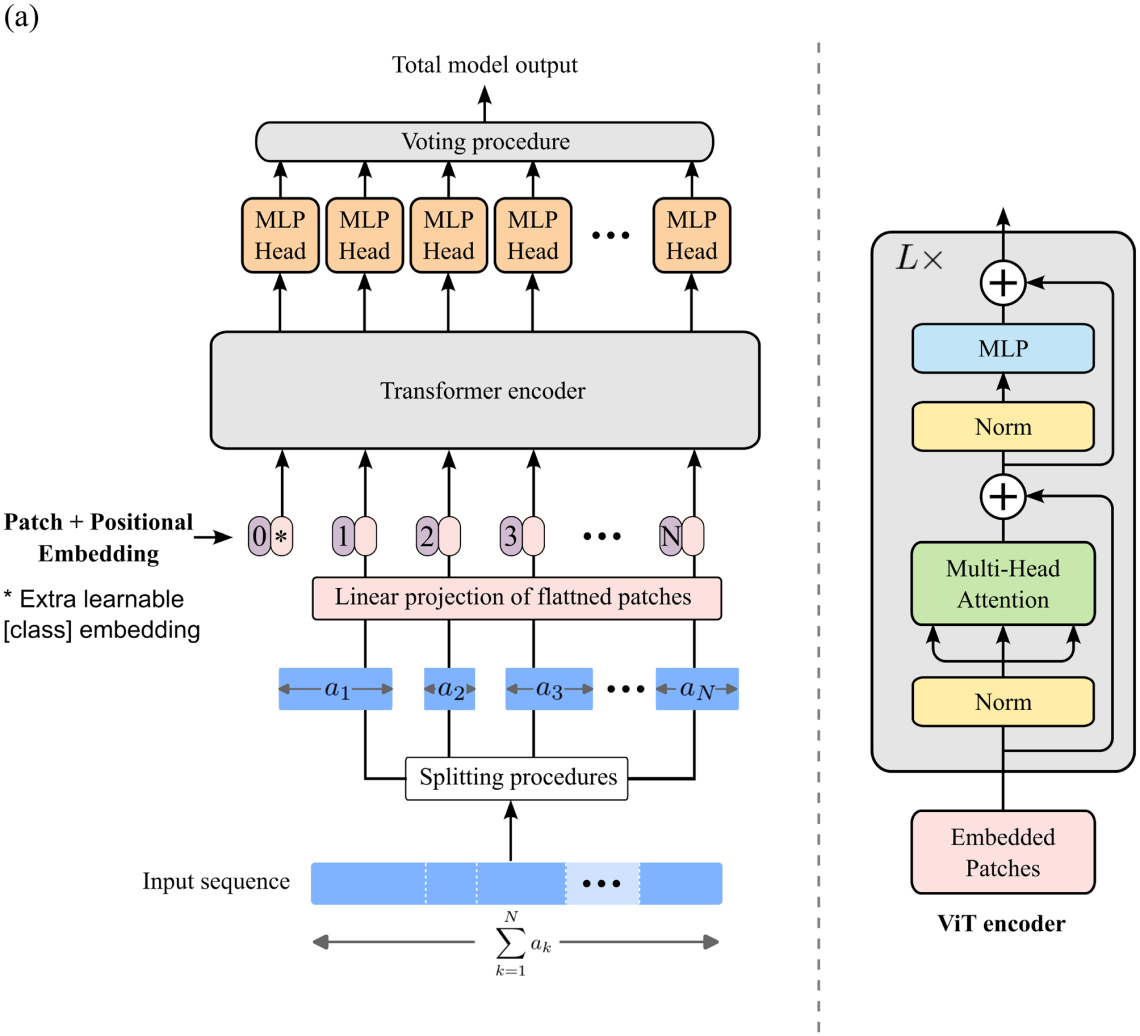

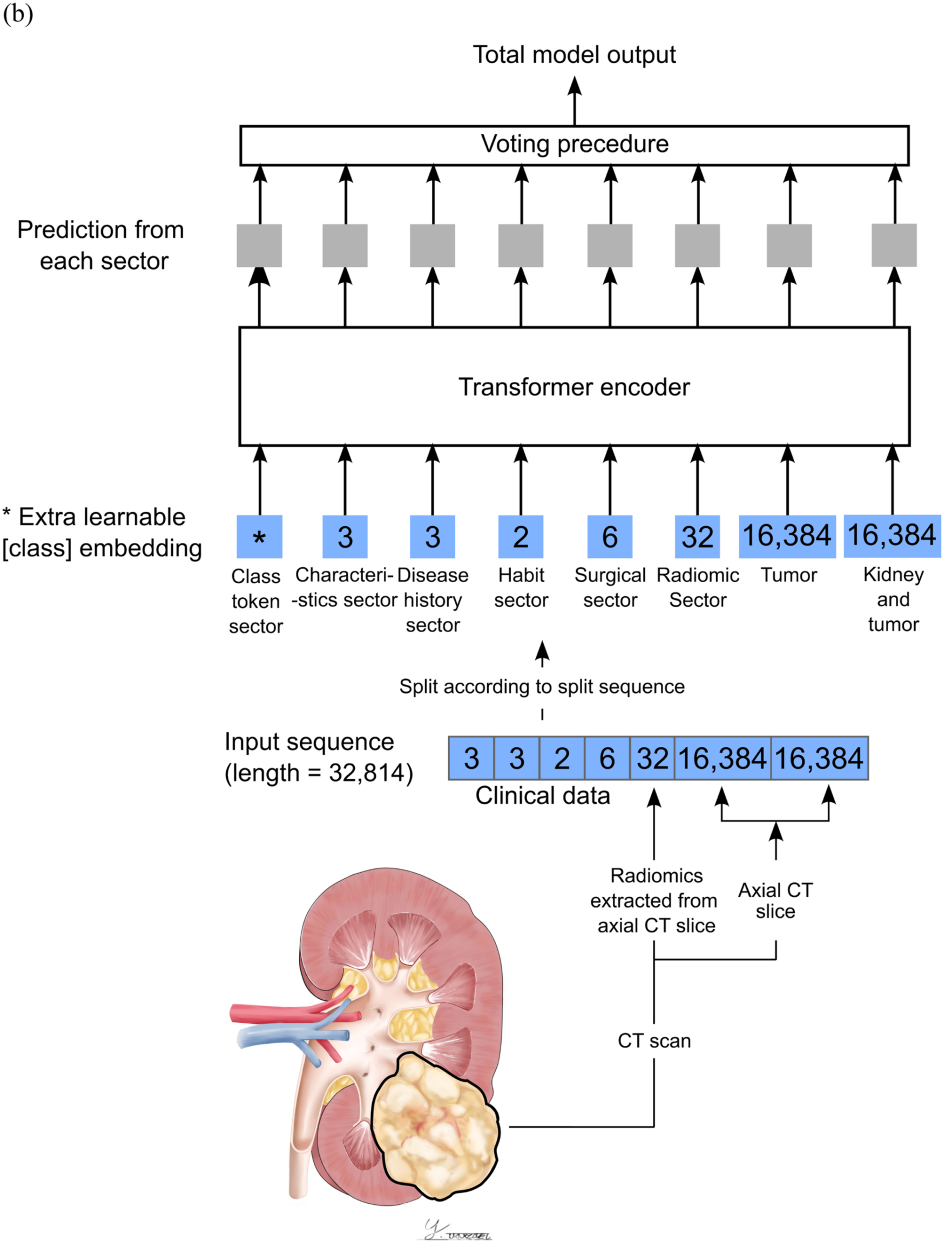
**a** Scheme of the variable vision transformer (vViT). The vViT receives input and split-sequence $\left\{{a}_{n}\right\}, 1\le n\le N$. The length of the input sequence is $\sum_{i=1}^{N}{a}_{i}$. The split sequences are then input to each sector, with the length of ${a}_{1}, {a}_{2}, \cdots , {a}_{N}$. Each sequence is input into the transformer encoder after concatenating the class token. Inspired by the original ViT encoder, the transformer is composed of a norm layer (Norm), multi-head attention layer, and multilayer perceptron (MLP). Finally, MLP head and voting procedures are performed on the probability tensor, and the total model output is obtained. **b** Scheme of the variable vision transformer (vViT) constructed in this study. The number in the blue rectangle represents the length of arrays input into vViT; demographics (2 features; age, sex, and BMI), comorbidity sector (3 features; peripheral vascular disease, diabetes mellitus, and liver disease), habit sector (2 features; smoking history and alcohol intake), surgical sector (6 features; ischemia time, estimated blood loss, surgical type, surgical procedure, surgical approach, and operative time), radiomic sector, tumor image sector, and kidney and tumor sector. All arrays were converted to 1-dimensional arrays before inputting to vViT. The gray rectangle represents the prediction from each sector. The total model output was obtained by voting. Based on the original vViT we attached the class token sector. Abbreviations: CT: computed tomography

Figure 1a shows an overview of the vViT model architecture. The vViT receives a one-dimensional input sequence that undergoes a splitting procedure based on split-sequence. After division, the smaller split sequences are input into the Transformer encoder. The Transformer encoder comprises alternating layers of multiheaded self-attention and multilayer perceptron (MLP) blocks. Layer norm is applied before every block, and residual connections are established after each block. Our implementation of the MLP involved two layers with a Gaussian Error Linear Unit (GELU) non-linearity. Specifically, the patch dimension, head dimension, number of heads, MLP dimension, and depth were set to 32, 64, 2, 64, and 8, respectively. The classification head is realized through an MLP with four hidden layers and the SoftMax function. Each component of the Transformer and the classification head is termed a sector. Consequently, vViT can accommodate an arbitrary number of sectors that receive sequences of varying lengths. We added a class token to the input. The probability tensor, containing output from each sector, was integrated by voting into the total model output. In this integration, the respective probabilities of eGFR deterioration in all sectors were averaged. The total model output was determined using these averaged probabilities. In vViT, the output of each sector is derived from the probability tensor. Each sector was named according to the property of its input, such as class token sector, demographics sector, comorbidity sector, habit sector, radiomic sector, tumor image sector, or kidney and tumor sector. Pytorch version 1.7.1 was used to implement vViT as the deep learning framework. Binary cross-entropy was optimized by the Adam optimizer (β1 = 0.9, β2 = 0.999, ε = 1.0 × 10–8, weight decay = 0, AMSGrad = False). The number of epochs was set to 200 for each model, and the parameters with the highest accuracy were saved.

### Radiomic Feature Extraction and Image Preprocessing

We extracted 105 radiomic features (Supplement 1) using the PyRadiomics package [12], and then derived two image types: tumor, and kidney with tumor. Each tumor was cropped by the minimum rectangles that contained the tumor. Similarly, kidney with tumor was cropped by the minimum rectangles that contained the kidney and tumor. The cropped image was expanded to a 128 × 128 image by the Python Pillow package with the LANCZOS option.

### Constructed Models

We constructed two models: vViT to predict patients whose eGFR decreased over 10 mL/min/1.73 m^2^ after surgical treatment (eGFR10 model), and vViT to predict patients whose eGFR decreased over 20 mL/min/1.73 m^2^ after surgical treatment (eGFR20 model). We imposed the same criteria on both models as described in the ***selection criteria***. In patients who underwent RN, there was no ischemia time for the residual kidney, and ischemia time was therefore considered and calculated as 0.

#### eGFR10 Model

**Fig. 2:**
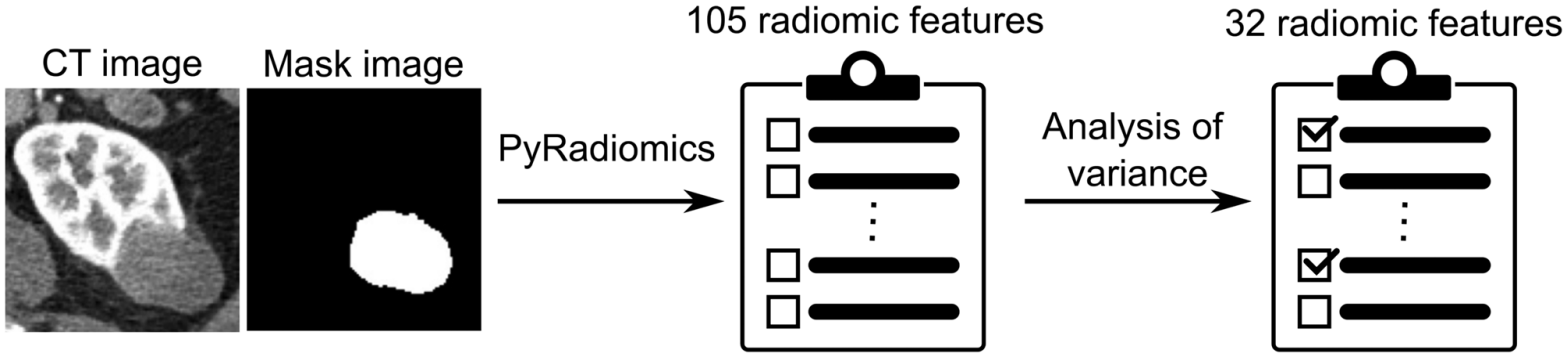
Radiomic Feature Extraction and Selection Process. The procedures of radiomic feature extraction and selection. A pair of computed tomography (CT) and mask images is need to extract radiomic features by PyRadiomics. After extracting 105 radiomic features, 32 radiomic features were selected using analysis of variance

The eGFR10 model was designed to classify patients whose eGFR reduction was over 10 (ΔeGFR > 10) and other patients (ΔeGFR ≤ 10) across the following eight sectors: class token sector, characteristics sector (age, sex, and BMI), comorbidity sector (peripheral vascular disease, diabetes mellitus, and liver disease), habit sector (smoking history and alcohol intake), surgical sector (ischemia time, estimated blood loss, surgical type, surgical procedure, surgical approach, and operative time), radiomic sector, tumor image sector, and kidney and tumor sector. Figure 1b shows the model summary constructed in this paper. Thirteen patients were excluded based on criteria (i) (due to missing ischemia time: 10, estimated blood loss: 1, and operative time: 2). Eighty-five patients were excluded by criteria (ii) (due to missing preoperative eGFR: 54 and postoperative eGFR: 31). Three patients were excluded by criterion (iii) due to insufficient pixel count, and one after applying criterion (iv), leaving 109 patients. Following random selection (v), seven patients were removed, resulting in a final total of 102 patients with 1,682 images (ΔeGFR > 10, n = 841; ΔeGFR ≤ 10, n = 841). Following the exclusion criteria, we divided the dataset into training and testing groups on a per-patient basis, ensuring each set respects the correlation of all images within individual cases. These patients were divided into a training dataset (75 patients with 1,340 images [ΔeGFR > 10, n = 670; ΔeGFR ≤ 10, n = 670]) and a test dataset (27 patients with 342 images [ΔeGFR > 10, n = 171; ΔeGFR ≤ 10, n = 171]). The selection processes are shown in Supplementary Fig. 1a. An analysis of variance was conducted to identify the radiomic features associated with eGFR decrease. The top 32 radiomic features by F-value were selected in descending order from the training dataset (Supplementary Table 1a). Figure 2 shows the radiomic feature selection process.

**Table 1:** Patient characteristics

Parameter	eGFR10			eGFR20		
	Training	Test	p-value	Training	Test	p-value
Number of patients	75	27	-	77	24	-
Number of images	1,340	342	-	778	236	-
Preoperative eGFR (95%CI), mL/min/1.73 m^2^	73.9 (70.5–77.2)	76.8 (72.3–81.3)	0.53	74.8 (71.7–77.9)	73.5 (67.3–79.7)	0.70
Postoperative eGFR (95%CI), mL/min/1.73 m^2^	61.1 (56.9–65.3)	67.0 (59.8–72.4)	0.14	63.0 (58.8–67.2)	60.5 (53.0–68.1)	0.57
Decreased by 10 or more, n (%)	40 (53.3)	14 (51.9)	0.74			-
Decreased by 20 or more, n (%)			-	24 (31.2)	9 (37.5)	0.67
Mean age (95%CI), years	61.1 (58.5–63.7)	59.9 (56.3–63.6)	0.59	61.0 (58.6–63.4)	60.8 (55.9–65.6)	0.93
Sex, n (%)			-			-
Male	42 (56.0)	17 (63.0)	0.80	45 (58.4)	13 (54.2)	0.21
Female	33 (44.0)	10 (37.0)	0.80	32 (41.6)	11 (45.8)	0.21
Mean BMI (95%CI), kg/m^2^	30.4 (29.0–31.9)	32.3 (29.6–35.0)	0.23	30.4 (29.2–31.7)	32.7 (29.0–36.3)	0.24
Peripheral vascular disease, n (%)	3 (4.0)	1 (3.7)	1.00	3 (39.0)	1 (4.2)	1.00
Liver disease, n (%)	1 (13.3)	0 (0.0)	1.00	1 (13.0)	0 (0.0)	1.00
Diabetes mellitus, n (%)	11 (14.7)	4 (14.8)	0.29	13 (16.9)	2 (8.3)	1.00
Smoking history, n (%)			-			
Current smoker	11 (14.7)	5 (18.5)	0.40	12 (15.6)	3 (12.5)	0.71
Previous smoker	31 (41.3)	15 (55.6)	0.44	33 (42.9)	13 (54.2)	0.71
Never smoked	33 (44.0)	7 (25.9)	0.44	32 (41.6)	8 (33.3)	1.00
Alcohol use, n (%)			-			
More than two drinks daily	5 (6.7)	2 (7.4)	0.89	4 (5.2)	2 (8.3)	0.50
Two or fewer drinks daily	43 (57.3)	16 (59.3)	0.72	46 (59.7)	13 (54.2)	1.00
Never or not in last 3 months	27 (36.0)	9 (33.3)	0.89	27 (35.1)	9 (37.5)	1.00
Mean ischemia time (95%CI), min	19.3 (16.1–22.4)	22.8 (14.6–31.1)	0.89	19.4 (15.7–23.0)	22.2 (16.3–28.0)	0.23
Mean estimated blood loss (95%CI), ml	309.2 (217.8–400.6)	360.2 (235.9–484.5)	0.50	354.8 (260.7–448.9)	231.0 (148.6–313.3)	0.048
Mean operative time (95%CI), min	248.5 (221.3–275.7)	251.4 (212.6–290.3)	0.90	255.6 (228.7–282.5)	226.1 (186.9–265.3)	0.21
Surgery type, n (%)			-			-
Robotic	43 (57.3)	17 (63.0)	1.00	47 (61.0)	12 (50.0)	0.68
Open	19 (25.3)	6 (22.2)	0.78	18 (23.4)	7 (29.2)	1.00
Laparoscopic	13 (17.3)	4 (14.8)	0.78	12 (15.6)	5 (20.8)	0.68
Surgical procedure, n (%)			-			-
Partial nephrectomy	45 (60.0)	18 (66.7)	0.088	50 (64.9)	12 (50.0)	0.64
Radical nephrectomy	30 (40.0)	9 (33.3)	0.088	27 (35.1)	12 (50.0)	0.64
Surgical approach, n (%)			-			-
Transperitoneal	63 (84.0)	24 (88.9)	1.00	65 (84.4)	21 (87.5)	1.00
Retroperitoneal	12 (16.0)	3 (11.1)	1.00	12 (15.6)	3 (12.5)	1.00

*95%CI* 95% confidence interval, *BMI* body mass index, *RCC* renal cell carcinoma, *eGFR* estimated glomerular filtration rate

#### eGFR20 Model

The eGFR20 model was designed to classify patients whose eGFR reduction was over 20 (ΔeGFR > 20), and other patients (ΔeGFR ≤ 20) across the same seven sectors in the eGFR10 model. Thirteen patients were excluded based on criteria (i) (due to missing ischemia time: 10, estimated blood loss: 1, and operative time: 2). Eighty-five patients were excluded by criteria (ii) (due to missing preoperative eGFR: 54 and postoperative eGFR: 31). One patient was excluded by criterion (iii) due to insufficient pixel count, and two patients were excluded after applying criterion (iv), leaving 109 patients. Following random selection (v), one patient was removed, resulting in a final total of 101 patients with 1,014 images (507 ΔeGFR > 20, 507 ΔeGFR ≤ 20). These patients were divided into a training dataset (77 patients with 778 images [389 ΔeGFR > 20, 389 ΔeGFR ≤ 20]) and a test dataset (24 patients with 236 images [118 ΔeGFR > 20, 118 ΔeGFR ≤ 20]). As in the eGFR10 model case, we divided the dataset into training and testing groups on a per-patient basis, ensuring each set respects the correlation of all images within individual cases. The selection processes are shown in Supplementary Fig. 1b. An analysis of variance was conducted to select radiomic features associated with eGFR decrease. The top 32 radiomic features by F-value were selected in descending order from the training dataset (Supplementary Table 1b). The both eGFR10 and eGFR20 models had the same architecture.

### Statistical Analysis

The characteristics of the patients and the calculated values are presented as the mean and 95% confidence interval (95%CI) or as the number (n) and ratio (%). The differences between the train and test datasets were tested using the Mann–Whitney U test for continuous variables and the chi-square test for categorical variables. As vViT was implemented to classify the patients into those in whom eGFR decreased and other patients, using images (image-based analysis), we organized the output of vViT to provide predictions for each patient (patient-based analysis). In this organizational approach, voting and mean were used for binary and continuous variables, respectively. We calculated the metrics of classification accuracy, sensitivity, specificity, positive predictive value (PPV), negative predictive value (NPV), F-score, the area under the curve of receiver-operating characteristics (AUC-ROC), logarithmic loss, and kappa score for evaluating the performance of vViT. To evaluate the contribution of each sector to output, permutation feature importance was calculated by performing the following three calculation steps.

(i) Permutation was performed to the respective sector according to the method reported previously [13]. By this implementation, data of a patient was assigned to another patient.
(ii) After permutation was performed, accuracy was calculated using trained vViT.
(iii) Then, the difference between the original accuracy and the accuracy calculated using the permutated dataset was saved.

**Fig. 3:**
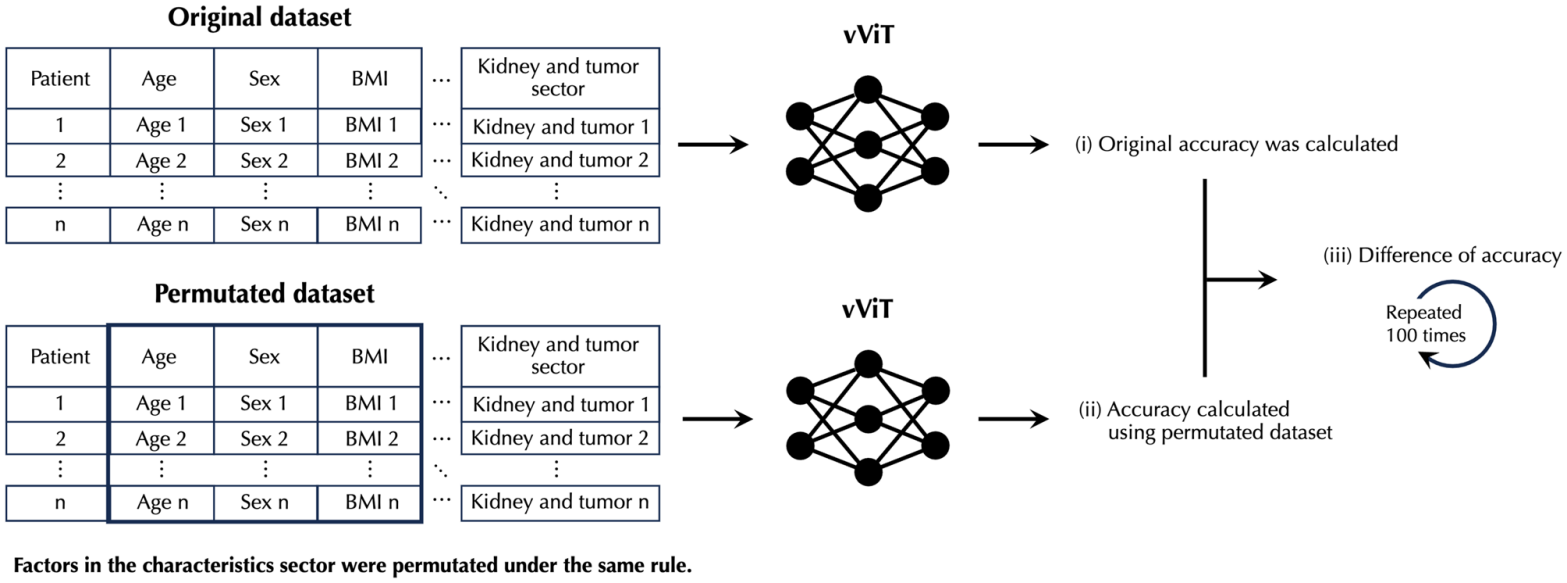
Procedures of permutation importance analysis. The characteristics sector is permutated as an example. The following three steps were performed. **i** The original accuracy was calculated using the original dataset and trained variable vision transformer (vViT). **ii** Permutation was then performed, and accuracy was calculated using a permutated dataset and trained vViT. **iii** The difference between the original accuracy and the accuracy calculated using a permutated dataset was calculated. These processes were repeated 100 times by changing the permutation pattern

Procedures (i), (ii), and (iii) were repeated 100 times for each sector. Figure 3 shows the procedures for calculating difference of accuracy, using the characteristics sector as an example. We compared the difference of accuracy in each sector using the Mann–Whitney U test. The performance of vViT model was compared with convolutional neural network (CNN) models; visual geometry group (VGG) 16 [19], Residual Networks (ResNet) 50 [20], and Dense Convolutional Network (DenseNet) 121 [21]. These models were tested after 1000 epochs training using each tumor and kidney and tumor images. We used the same training and test datasets as used for vViT. McNemar test and DeLong test were performed to compare contingency table and AUC-ROC, respectively. All analyses were performed using Python Language, version 3.8.2 (Python Software Foundation at http://www.python.org). Statistical significance was evaluated by 95%CI and p-value < 0.05.

## Results

Table 1 shows the characteristics of patients in the eGFR10 and eGFR20 models.

### eGFR10 Model

**Fig. 4:**
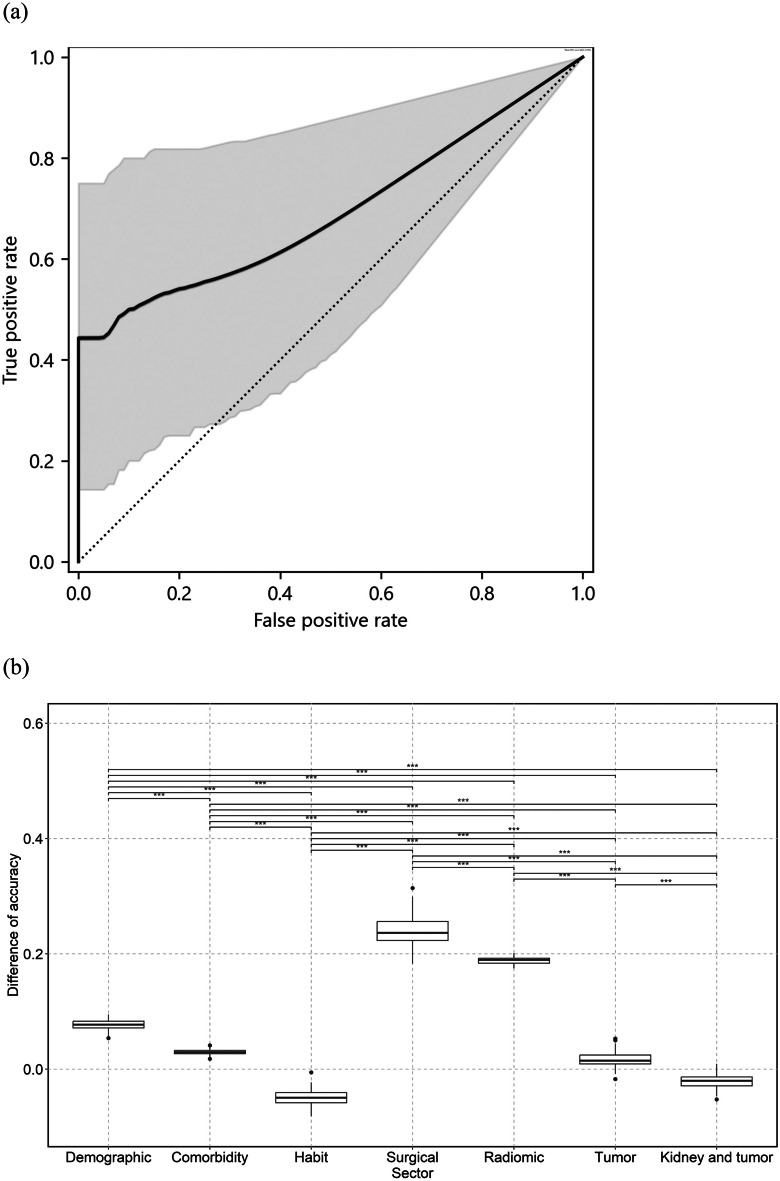

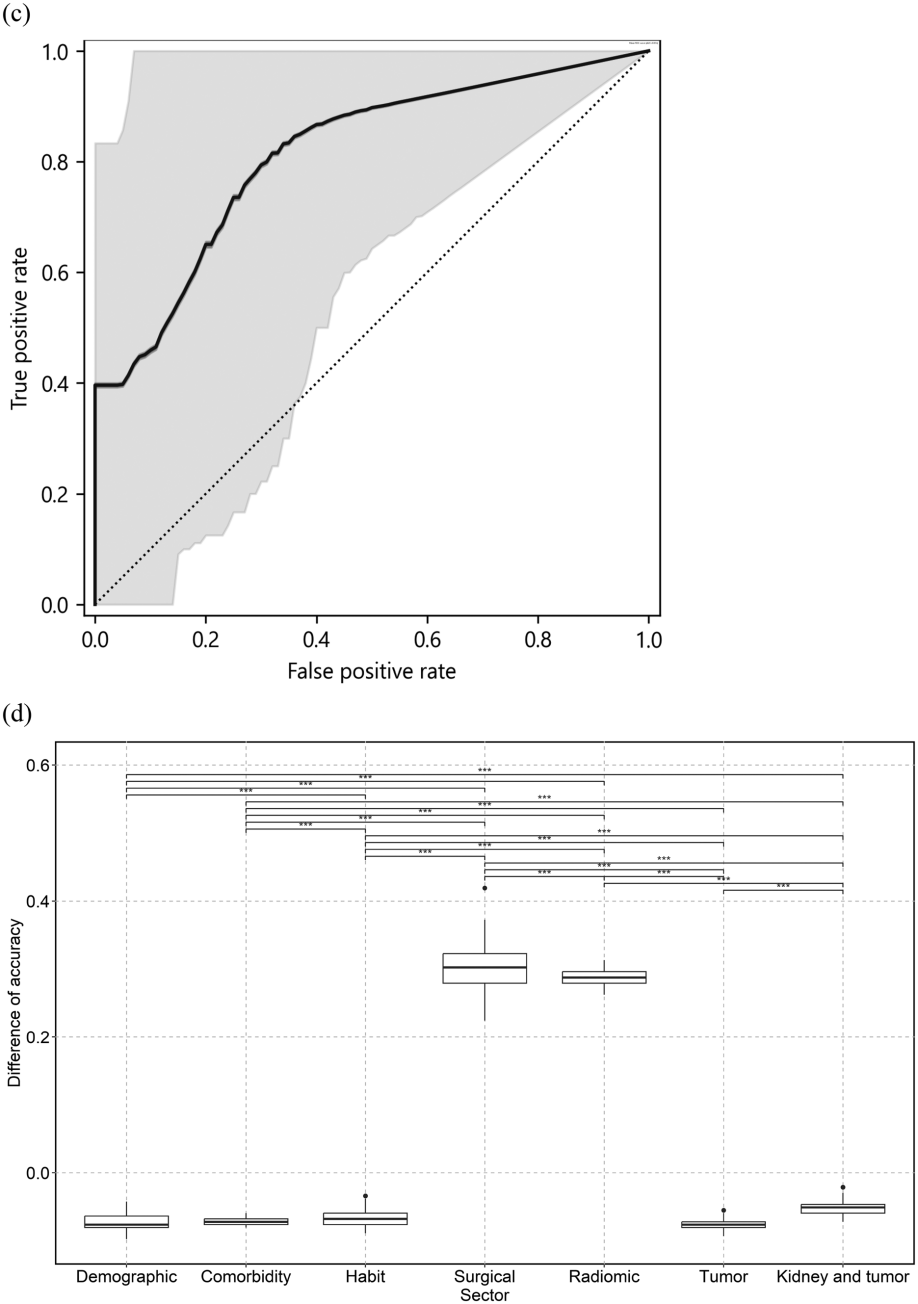
Receiver-operating characteristic curve and results of permutation importance analysis for the test dataset. Curves of receiver-operating characteristics (ROC) for image-based analysis and patient-based analysis of the test dataset are shown in (**a**) and (**c**), respectively. The gray zone in each figure represents the 95% confidence interval (95% CI). Box plots illustrating the difference between the original accuracy and the accuracy calculated with the permuted dataset for respective sectors in the eGFR10 and eGFR20 model patient-base analyses are shown in (**b**) and (**d**), respectively. The horizontal line of the upper whisker, the upper horizontal line of the rectangle, the gray line, the lower horizontal line of the rectangle, and the horizontal line of the lower whisker represent the maximum, third quartile, median, first quartile, and minimum, respectively. Bars on boxplots show the results of the Mann–Whitney U test. * p < 0.05, ** p < 0.001, and *** p < 0.0001

Accuracy, sensitivity, specificity, PPV, NPV, F-score, AUC-ROC, logarithmic loss, and kappa score for the test dataset were 0.741 (95%CI; 0.615–0.806), 0.538 (0.375–0.684), 0.929 (0.716–0.956), 0.875 (0.574–0.933), 0.684 (0.532–0.774), 0.667 (0.489–0.844), 0.692 (0.489–0.861), 0.555 (0.504–0.607), and 0.474 (0.392–0.555), respectively. Sectors ranked in descending order of importance of permutation feature are as follows: surgical (difference between the original accuracy and accuracy calculated using permuted dataset = 0.249, 95%CI; 0.248–0.251), radiomics (0.234, 0.233–0.236), comorbidity (0.113, 0.111–0.114), demographics (0.0858, 0.0841–0.0875), habit (0.0336, 0.0319–0.0352), kidney and tumor (–0.0211, –0.0228 to –0.0194), and tumor (–0.0250, –0.0267 to –0.0234) sectors. Figure 4a and b show the ROC and the permutation feature importance of each sector for the test dataset, respectively. When using tumor images, vViT did not have a statistically different contingency table from VGG16 (p-value = 0.34) and ResNet50 (1.0) and had a statistically different contingency table from DenseNet121 (0.0010). There was no statistical difference in AUC-ROC compared with VGG16 (1.0), ResNet50 (0.70), and DenseNet121 (0.87). When using tumor and kidney images, the vViT did not have a statistically different contingency table from VGG16 (0.057) and had a statistically different contingency table from ResNet50 (0.00030) and DenseNet121 (0.021). There was no statistical difference in AUC-ROC compared with VGG16 (0.73), ResNet50 (0.91), and DenseNet121 (0.81). Tables 2a and 3a list the statistical data for each sector and the results of the comparison with the CNN models, respectively. The results of the image-based analysis are shown in Supplement 2a, Supplementary Fig. 2a, b, Supplementary Tables 2a, and 3a.

### eGFR20 Model

Accuracy, sensitivity, specificity, PPV, NPV, F-score, AUC-ROC, logarithmic loss, and kappa score for the test dataset were 0.792 (95% CI; 0.653–0.850), 0.875 (0.574–0.933), 0.750 (0.572–0.831), 0.636 (0.434–0.768), 0.923 (0.700–0.954), 0.737 (0.561–0.913), 0.812 (0.609–0.972), 1.29 (1.22–1.36), and 0.571 (0.492–0.650), respectively. Sectors ranked in descending order of importance of permutation feature are as follows: surgical (difference between the original accuracy and accuracy calculated using permuted dataset = 0.302, 95%CI; 0.299–0.305), radiomics (0.288, 0.285–0.291), kidney and tumor (–0.0513, –0.0541 to –0.0485), habit (–0.0664, –0.0692 to –0.0635), comorbidity (–0.0710, –0.0738 to –0.0682), demographics (–0.0741, –0.0769 to –0.0712), and tumor (–0.0757, –0.0785 to –0.0729) sectors. Figure 4c and d show the ROC and the permutation feature importance of each sector for the test dataset, respectively. When using tumor images, vViT did not have a statistically different contingency table from VGG16 (p-value = 0.18), ResNet50 (0.79), and DenseNet121 (0.58). There was no statistical difference in AUC-ROC compared with VGG16 (0.72), ResNet50 (0.88), and DenseNet121 (0.64). When using tumor and kidney images, vViT did not have a statistically different contingency table from VGG16 (p-value = 0.34), ResNet50 (0.092), and DenseNet121 (0.39). There was no statistical difference in AUC-ROC compared with VGG16 (0.34), ResNet50 (0.94), and DenseNet121 (0.11). Tables 2b and 2b list the statistical data for each sector and the results of the comparison with the CNN models, respectively. The results of the image-based analysis are shown in Supplement 2b, Supplementary Fig. 2c, d, Supplementary Tables 2a, and  3b.

## Discussion

This study aims to evaluate the effectiveness of vViT in predicting postoperative renal function decline using clinical information, medical images, and derived image features. We used preoperative information to predict and tried to identify the primary factor influencing on the deterioration. To the best of our knowledge, this is the first application of vViT for multimodal analysis of postoperative renal function, particularly for identifying the dominant factor among demographics, radiomic features, and CT imaging. In the eGFR10 model test dataset, vViT showed accuracy of 0.741 and AUC-ROC of 0.692. For the eGFR20 model, accuracy was 0.792 and AUC-ROC was 0.812. Since the eGFR10 and eGFR20 models had the same architecture, the difference in performance just reflected the difficulty level of tasks. Although vViT achieved comparable performance with CNN models, vViT did not achieve state-of-the-art performance. Kuo et al. achieved higher accuracy (0.86) and AUC-ROC (0.90) in their study of CKD prediction using deep learning and ultrasound images [6]. Notably, their accuracy surpassed that of experienced nephrologists (0.603–0.801) [6]. Although ultrasound is convenient and non-invasive, its results are operator-dependent [14], whereas CT provides more consistent and reproducible outcomes. Direct comparison with the present study is challenging because they used a different definition of CKD (eGFR < 60 mL/min/1.73 m^2^).

The eGFR10 model's two most important sectors were the surgical (permutation feature importance = 0.249, 95%CI: 0.248–0.251) and radiomic (0.234, 0.233–0.236) sectors. Similarly, the eGFR20 model's two most important features were the surgical (0.302, 0.299–0.305) and radiomic (0.288, 0.285–0.291) sectors. Especially in clinical practice, we should consider surgical information (ischemia time, estimated blood loss, surgical type, surgical procedure, surgical approach, and operative time). A previous study showed that surgical factors such as ischemic time and type of surgery had an impact on predicting a decline in eGFR [15]. In cases where surgical intervention is complex or extensive, surgical factors may influence the prediction of future decline in kidney function. Radiomics analysis, in which quantitative features are extracted from medical images, provides comprehensive data on kidney and tumor characteristics [16]. Radiomic features provide detailed insight into the pathology of the kidney and associated tumors [16]. In a previous study, Shimada et al. identified preoperative eGFR and age as significant predictors of deteriorating renal function after RN in both males and females, whereas tumor size and BMI were significant predictors only in males [5]. The study of Ishiyama et al. showed that BMI and diabetes mellitus are associated with upstaging of kidney cancer [17]. Although the demographic sector had relatively less contribution than the surgical and radiomic sectors, they still played a role in prediction. In the present study, we input two types of images: tumor only and kidney and tumor. The properties of a kidney tumor should be discussed based on its association with normal tissue as well as the tumor itself. Radiomics cannot analyze the association between tumor and normal tissue because radiomic features are calculated to the region specified by a mask image. We included images of the kidney and tumor in addition to the tumor images. Our analyses revealed that CT images had a relatively lower contribution compared with surgical information and radiomic features. Combining CNN with vViT may be a solution. Another method that analyzes the association between tumor and normal tissue should be investigated. As a rationale behind the importance ranking, the sector with higher permutation importance had a higher contribution to the prediction. The permutation feature importance analysis assumes that random permutation of input data decreases the performance of a deep learning model. The development of effective multimodal fusion approaches is becoming increasingly important to capture features of complex diseases [18]. Predicting deterioration in postoperative renal function among adult patients is not an exception.

The performance of the vViT was compared to various CNN models using both the tumor, and kidney and tumor images. Our analyses revealed that, in the eGFR10 model, vViT had the potential to outperform ResNet50 and DenseNet. In addition to this, the non-inferiority of vViT compared with CNN models in AUC-ROC was proven for both eGFR10 and eGFR20 models.

The present study has some limitations. First, our study utilized a retrospective data collection method from a single center, which, after specific exclusions, might have introduced selection bias. We acknowledge the importance of external validation using diverse datasets to enhance the generalizability of our findings. However, the collection of additional datasets is challenging due to constraints. We recognize that our findings cannot be broadly generalized without further validation. Future research should focus on validating the generalizability of our results using additional datasets and exploring comparisons with radiologists or diagnostic improvements by radiologists using our model. Second, an inherent variability may be present due to potential differences in the timing of blood sampling among patients, which could have influenced the eGFR measurements. Following PN or RN, patients are at risk of deterioration of renal function due to the invasive nature of the surgical procedures. Some patients recover from the deterioration in function, but others do not. It is also necessary to consider the timing of blood sampling, which should be consistent among patients, e.g., the day after surgery and at the 3-month follow-up. Rigorous follow-up would clarify the time course of the deterioration of eGFR. In the present study, however, we analyzed patients at follow-up after discharge and the effect of the time of blood sampling may have been limited. Third, our analysis compared different sectors, but further studies using traditional statistical methods are required to pinpoint the most influential factors within each sector. Although we combined inputs from all sectors, a more in-depth analysis could offer clearer insights into their complex interactions. Focusing on radiomics and the CT image itself, we were able to compare these in one model. A medical image has full information, and it is doubtful that a deep-learning model will learn the appropriate area. On the other hand, radiomics is a statistic of a region of interest, and we lose information in other areas. We believe that vViT may be a solution to multimodal analysis for medical image analysis. Finally, we were unable to provide straightforward visualizations that effectively illustrate the model's decision-making process. While the importance of AI result visualization to enhance explainability is well-recognized, the visualization techniques for different model architectures vary. Grad-CAM, a popular method for visualizing CNN models, does not directly apply to transformer-based models due to fundamental differences in architecture. In transformer-based models like ViT, attention maps are typically used to interpret the model's focus areas. However, generating these visualizations cannot be directly applied to vViT. However, our study used segmented tumor images, which are designed to specify regions of interest. This design choice further complicates the visualization process, as it requires the model to focus on pre-defined areas rather than learning these areas autonomously. This limitation highlights the need for further research into developing robust visualization techniques for transformer-based models, particularly in medical imaging. Future work should focus on overcoming these challenges to improve the interpretability and explainability of such advanced AI models. These limitations should be considered in future research Table 3.

## Conclusion

In conclusion, vViT is an effective model for predicting postoperative renal function decline in adults. When combined with permutation feature importance, the surgical and radiomics sectors were the most influential factors in both the eGFR10 and eGFR20 models. The high permutation importance of these factors underscores their critical roles in this prediction.

## Supplementary Information

Below is the link to the electronic supplementary material.

Supplementary file1 (DOCX 1075 KB)

## Data Availability

The data used in this study can be obtained at  https://wiki.cancerimagingarchive.net/pages/viewpage.action?pageId=61081171. We are willing to distribute source code of vViT for those who are interested in accessing the source code. Please contact the corresponding author.

## Abbreviations

95% CI: Confidence interval
C4KC-KiTS: 2019 Kidney and Kidney Tumor Segmentation Challenge
AUC-ROC: Area under the curve of receiver-operating characteristics
BMI: Body mass index
CKD: Chronic kidney disease
CNN: Convolutional neural network
CT: Computed tomography
DenseNet121: Dense Convolutional Network 121
GELU: Gaussian error linear unit
eGFR: Estimated glomerular filtration rate.
MLP: Multilayer perceptron
NPV: Negative predictive value
PPV: Positive predictive value
RCC: Renal cell carcinoma
ResNet50: Residual Networks 50
VGG16: Visual geometry group 16
vViT: Variable vision transformer

## References

[1] Chandrasekar T, Boorjian SA, Capitanio U, Gershman B, Mir MC, Kutikov A. Collaborative Review: Factors Influencing Treatment Decisions for Patients with a Localized Solid Renal Mass. European Urology. 10.1016/j.eururo.2021.01.021. 10.1016/j.eururo.2021.01.021 33558091

[2] Mir MC, Derweesh I, Porpiglia F, Zargar H, Mottrie A, Autorino R. Partial Nephrectomy Versus Radical Nephrectomy for Clinical T1b and T2 Renal Tumors: A Systematic Review and Meta-analysis of Comparative Studies. European Urology. 10.1016/j.eururo.2016.08.060. 10.1016/j.eururo.2016.08.060 27614693

[3] Kim SP, Campbell SC, Gill I, Lane BR, Van Poppel H, Smaldone MC, Volpe A, Kutikov A. Collaborative Review of Risk Benefit Trade-offs Between Partial and Radical Nephrectomy in the Management of Anatomically Complex Renal Masses. European Urology. 10.1016/j.eururo.2016.11.038. 10.1016/j.eururo.2016.11.038 27988238

[4] Van Poppel H, Da Pozzo L, Albrecht W et al. A Prospective, Randomised EORTC Intergroup Phase 3 Study Comparing the Oncologic Outcome of Elective Nephron-Sparing Surgery and Radical Nephrectomy for Low-Stage Renal Cell Carcinoma. European Urology. 10.1016/j.eururo.2010.12.013. 10.1016/j.eururo.2010.12.013 21186077

[5] Shimada S, Saito H, Kawasaki Y et al. Clinical predictors of the estimated glomerular filtration rate 1 year after radical nephrectomy in Japanese patients. Investig Clin Urol. 2017 Jul;58(4):228-234. 10.4111/icu.2017.58.4.228. 28681031 10.4111/icu.2017.58.4.228 PMC5494345

[6] Kuo CC, Chang CM, Liu KT et al. Automation of the kidney function prediction and classification through ultrasound-based kidney imaging using deep learning. NPJ Digit Med 2:29. 10.1038/s41746-019-0104-2, April 26, 2019. 10.1038/s41746-019-0104-2 PMC6550224 31304376

[7] Usuzaki T. Splitting expands the application range of Vision Transformer – variable Vision Transformer (vViT). arXiv. 2023, 10.48550/ARXIV.2211.03992.

[8] Dosovitskiy A, Beyer L, Kolesnikov A et al. Weissenborn D, Zhai X, Unterthiner T, Dehghani M, Minderer M, Heigold G, Gelly S, Uszkoreit J, Houlsby N. An Image is Worth 16x16 Words: Transformers for Image Recognition at Scale. arXiv, 2021. 10.48550/ARXIV.2010.11929.

[9] Usuzaki T, Takahashi K, Inamori R et al. Grading diffuse glioma based on 2021 WHO grade using self-attention-base deep learning architecture: variable Vision Transformer (vViT). Biomedical Signal Processing and Control. 2024, 10.1016/j.bspc.2024.106001.

[10] Fahad S, Salman K, Syed W et al. Transformers in medical imaging: A survey. Medical Image Analysis. 2023, 10.1016/j.media.2023.102802. 10.1016/j.media.2023.102802 37315483

[11] Heller N, Sathianathen N, Kalapara A et al. The KiTS19 Challenge Data: 300 Kidney Tumor Cases with Clinical Context. CT Semantic Segmentations, and Surgical Outcomes. 2019. 10.48550/ARXIV.1904.00445.

[12] van Griethuysen JJM, Fedorov A, Parmar C et al. Computational Radiomics System to Decode the Radiographic Phenotype. *Cancer Res* 2017;77:e104-e107. 29092951 10.1158/0008-5472.CAN-17-0339 PMC5672828

[13] Usuzaki T, Ishikuro M, Murakami K et al. How can we evaluate whether an association is truly inter-generational? *J Hypertens* 2020;38:1866-1868 32769690 10.1097/HJH.0000000000002507

[14] M.A. Sargent, B.P.M. Wilson, Observer variability in the sonographic measurement of renal length in childhood, Clinical Radiology, 1992, 10.1016/S0009-9260(05)80382-4. 1464209 10.1016/s0009-9260(05)80382-4

[15] Liu X, Jin D, Zhang Y, Zhang S. Limited non-linear impact of warm ischemia time on renal functional decline after partial nephrectomy: a propensity score-matched study. Int Urol Nephrol. 2023 Jul;55(7):1699-1708. doi: 10.1007/s11255-023-03630-0. Epub 2023 May 16. PMID: 37191733. 37191733 10.1007/s11255-023-03630-0

[16] Suarez-Ibarrola R, Basulto-Martinez M, Heinze A, Gratzke C, Miernik A. Radiomics Applications in Renal Tumor Assessment: A Comprehensive Review of the Literature. Cancers (Basel). 2020 May 28;12(6):1387. doi: 10.3390/cancers12061387. PMID: 32481542; PMCID: PMC7352711. 32481542 10.3390/cancers12061387 PMC7352711

[17] Ishiyama R, Omae K, Kondo T et al. Predictive factors and oncological outcomes of pathological T3a upstaging in patients with clinical T1 renal cell carcinoma undergoing partial nephrectomy. Japanese Journal of Clinical Oncology, 2023, hyad142, 10.1093/jjco/hyad142. 10.1093/jjco/hyad142 37840320

[18] Steyaert, S., Pizurica, M., Nagaraj, D. et al. Multimodal data fusion for cancer biomarker discovery with deep learning. Nat Mach Intell 5, 351–362 (2023). 10.1038/s42256-023-00633-5 37693852 10.1038/s42256-023-00633-5 PMC10484010

[19] Simonyan K, Zisserman A: Very Deep Convolutional Networks for Large-Scale Image Recognition. arXiv, 10.48550/arXiv.1409.1556, September 4, 2014.

[20] He K, Zhang X, Ren S, Sun J: Deep Residual Learning for Image Recognition. CoRR, DOI: 10.48550/arXiv.1512.03385, December 10, 2015.

[21] Huang G, Liu Z, van der Maaten L, Weinberger KQ: Densely Connected Convolutional Networks. arXiv, 10.48550/arXiv.1608.06993, August 25, 2016.

[22] Takuma U, Ryusei I, Takashi S, Yohei M, Hidenobu T, Mami I, Taku O, Kei T (2024) Predicting isocitrate dehydrogenase status among adult patients with diffuse glioma using patient characteristics radiomic features and magnetic resonance imaging: Multi-modal analysis by variable vision transformer. Magnetic Resonance Imaging 1266-276. 10.1016/j.mri.2024.05.012 10.1016/j.mri.2024.05.012 38815636

[23] Takuma, Usuzaki Kengo, Takahashi Ryusei, Inamori Yohei, Morishita Takashi, Shizukuishi Hidenobu, Takagi Mami, Ishikuro Taku, Obara Kei, Takase (2024) Identifying key factors for predicting O6-Methylguanine-DNA methyltransferase status in adult patients with diffuse glioma: a multimodal analysis of demographics radiomics and MRI by variable Vision Transformer Abstract Neuroradiology 66(5) 761-773. 10.1007/s00234-024-03329-8 10.1007/s00234-024-03329-8 PMC11031474 38472373

