# Supplementary material for: Predicting EGFR Status After Radical Nephrectomy or Partial Nephrectomy for Renal Cell Carcinoma on CT Using a Self-attention-based Model: Variable Vision Transformer (vViT)
Source: J Imaging Inform Med. 2024 Jun 28;37(6):3057–69. doi: 10.1007/s10278-024-01180-0 (PMC11612086; doi:10.1007/s10278-024-01180-0)
Supplement: Supplementary file 1 — Supplementary file1 (DOCX 1075 KB) [file 10278_2024_1180_MOESM1_ESM.docx]

**Supplement 1**. The list of 105 radiomic features

First Order features (the number of features, 20)

The number of pixels, Energy, Total Energy, Entropy, Minimum, 10 Percentile, 90 Percentile, Maximum, Mean, Median, Range, Interquartile Range, Mean Absolute Deviation, Robust Mean Absolute Deviation, Root Mean Squared, Standard Deviation, Skewness, Kurtosis, Variance, and Uniformity.

Shape2D features (10)

Mesh Surface, Pixel Surface, Perimeter, Perimeter Surface Ratio, Sphericity, Spherical Disproportion, Maximum Diameter, Major Axis Length, Minor Axis Length, and Elongation.

Gray-Level Co-occurrence Matrix (GLCM) features (24)

Autocorrelation, Joint Average, Cluster Prominence, Cluster Shade, Cluster Tendency, Contrast, Correlation, Difference Average, Difference Entropy, Difference Variance, Joint Energy, Joint Entropy, Informational Measure of Correlation 1, Informational Measure of Correlation 2, Inverse Difference Moment, Maximal Correlation Coefficient, Inverse Difference Moment Normalized, Inverse Difference, Inverse Difference Normalized, Inverse Variance, Maximum Probability, Sum Average, Sum Entropy, and Sum Squares.

Gray Level Size Zone Matrix (GLSZM) features (16)

Small Area Emphasis, Large Area Emphasis, Gray Level Non Uniformity, Gray Level Non Uniformity Normalized, Size Zone Non Uniformity, Size Zone Non Uniformity Normalized, Zone Percentage, Gray Level Variance, Zone Variance, Zone Entropy, Low Gray Level Zone Emphasis, High Gray Level Zone Emphasis, Small Area Low Gray Level Emphasis, Small Area High Gray Level Emphasis, Large Area Low Gray Level Emphasis, and Large Area High Gray Level Emphasis.

Gray Level Run Length Matrix (GLRLM) features (16)

Short Run Emphasis, Long Run Emphasis, Gray Level Non Uniformity, Gray Level Non Uniformity Normalized, Run Length Non Uniformity, Run Length Non Uniformity Normalized, Run Percentage, Gray Level Variance, Run Entropy, Run Variance, Low Gray Level Run Emphasis, High Gray Level Run Emphasis, Short Run Low Gray Level Emphasis, Short Run High Gray Level Emphasis, Long Run Low Gray Level Emphasis, and Long Run High Gray Level Emphasis.

Neighboring Gray Tone Difference Matrix (NGTDM) features (5)

Busyness, Coarseness, Complexity, Contrast, and Strength.

Gray Level Dependence Matrix (GLDM) features (14)

Small Dependence Emphasis, Large Dependence Emphasis, Gray Level Non Uniformity, Dependence Non Uniformity, Dependence Non Uniformity Normalized, Gray Level Variance, Dependence Variance, Dependence Entropy, Low Gray Level Emphasis, High Gray Level Emphasis, Small Dependence Low Gray Level Emphasis, Small Dependence High Gray Level Emphasis, Large Dependence Low Gray Level Emphasis, and Large Dependence High Gray Level Emphasis.

**Supplementary Figure 1.** The number of patients and images excluded by exclusion criteria and random selection. (a) shows an overview of the eGFR10 model, and (b) shows an overview of the eGFR20 model.

Abbreviations: eGFR, estimated glomerular filtration rate.

1. eGFR10 model


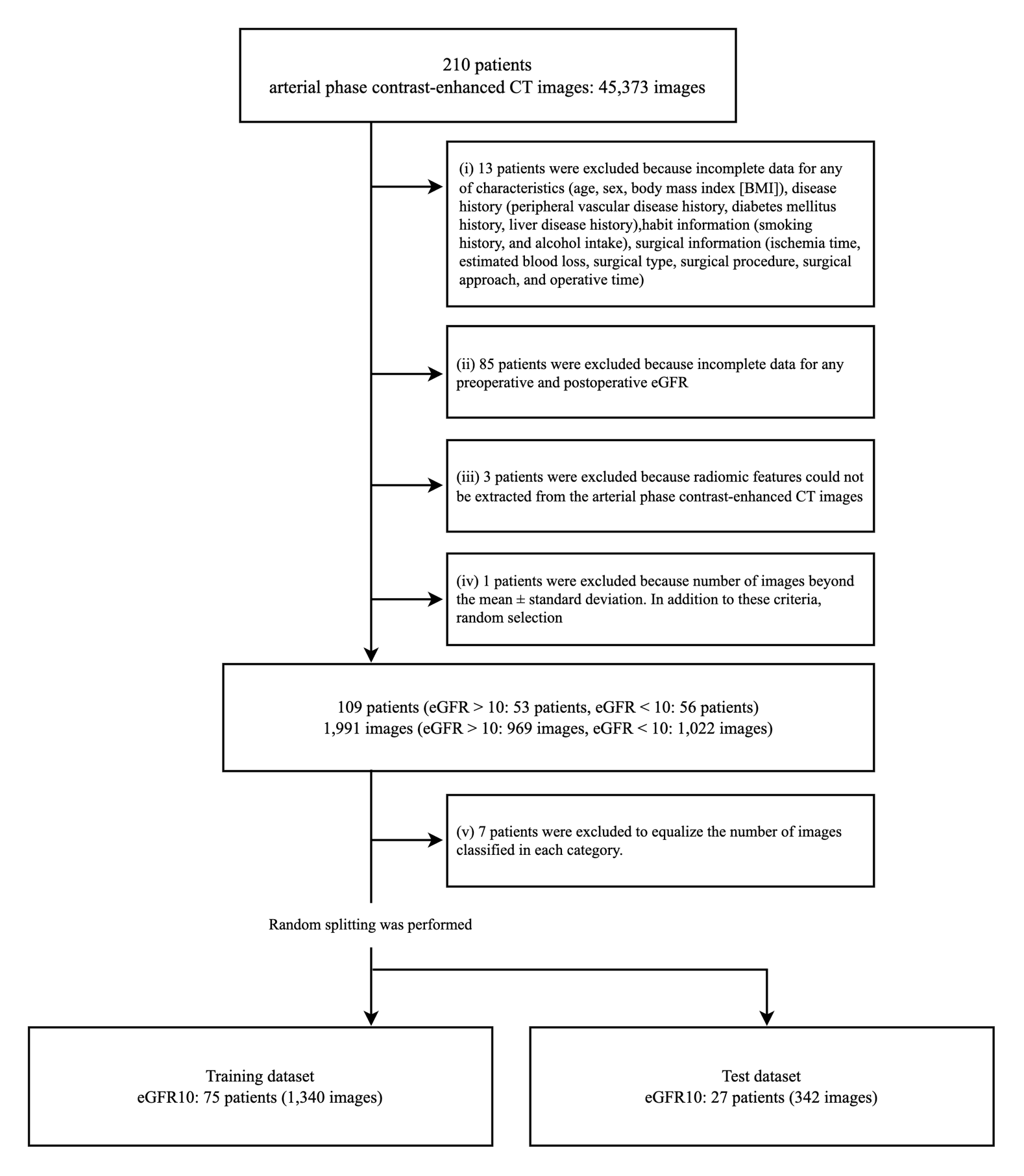


1. eGFR20 model


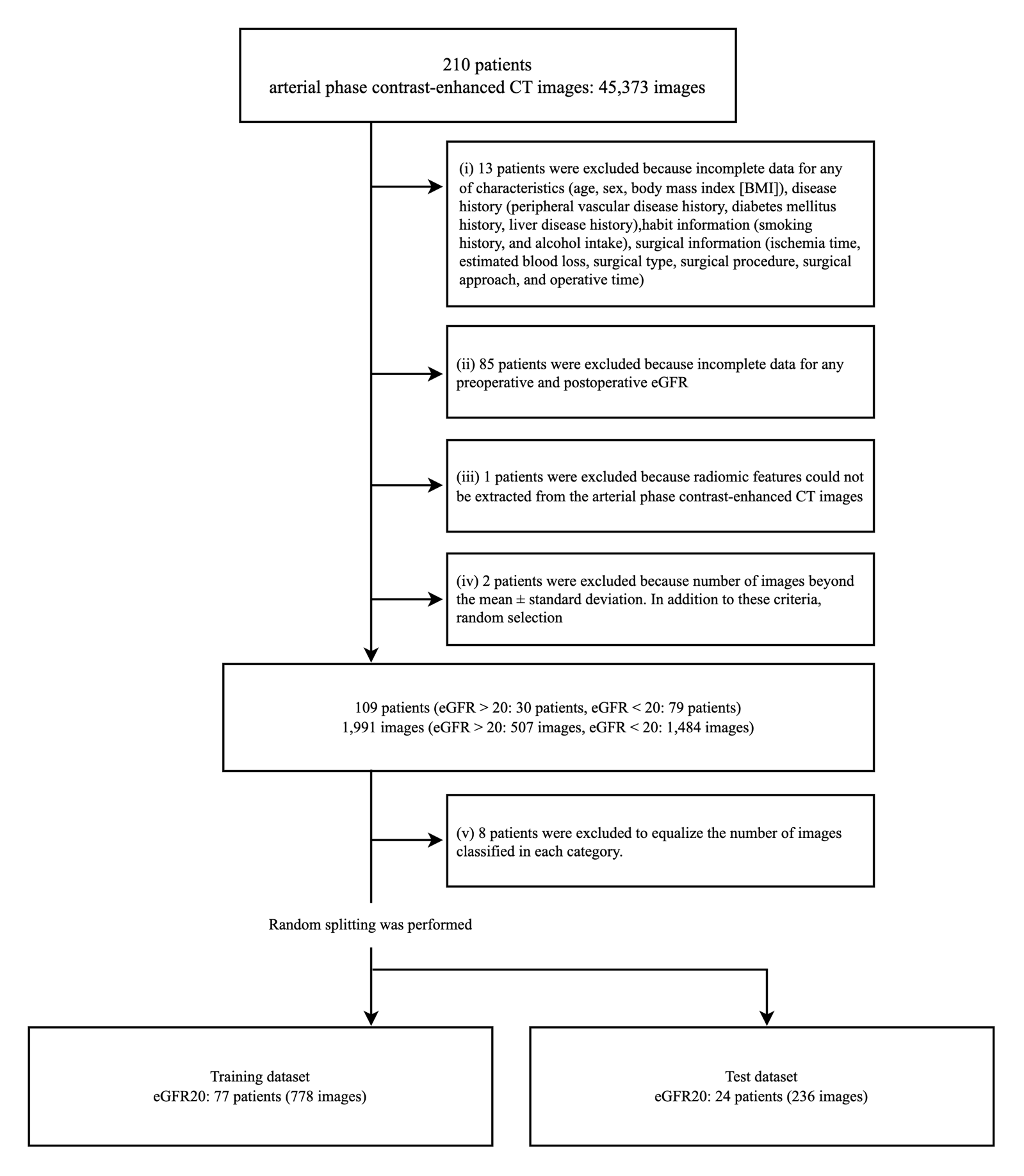


**Supplementary Table 1.**

(a) The list of selected radiomic features, F-value, and p-value for eGFR10 model

| model | Feature class | Feature name | F-value | p-value |
| --- | --- | --- | --- | --- |
| eGFR10 | First Order | Minimum | 126.77 | <0.0001 |
|  |  | 10Percentile | 34.03 | <0.0001 |
|  |  | 90Percentile | 74.86 | <0.0001 |
|  |  | Mean | 120 | <0.0001 |
|  |  | Median | 106.74 | <0.0001 |
|  |  | Range | 119.86 | <0.0001 |
|  |  | RootMeanSquared | 126.46 | <0.0001 |
|  | Shape2D | Perimeter | 43.47 | <0.0001 |
|  |  | Perimeter Surface Ratio | 112.98 | 0.0002 |
|  |  | Sphericity | 38.21 | <0.0001 |
|  |  | Spherical Disproportion | 275.04 | <0.0001 |
|  |  | Maximum Diameter | 41.55 | <0.0001 |
|  |  | Major Axis Length | 348.57 | <0.0001 |
|  |  | Minor Axis Length | 282.99 | 0.0002 |
|  |  | Elongation | 252.93 | <0.0001 |
|  | GLCM | Idmn | 84.9 | 0.0068 |
|  | GLSZM | Small Area Emphasis | 31.65 | <0.0001 |
|  |  | Gray Level Nonuniformity | 60.97 | <0.0001 |
|  |  | Size Zone Nonuniformity | 54.49 | <0.0001 |
|  |  | Size Zone Nonuniformity Normalized | 70.81 | <0.0001 |
|  |  | High Gray Level Zone Emphasis | 123.1 | 0.0030 |
|  |  | Small Area High Gray Level Emphasis | 40.77 | <0.0001 |
|  | GLRLM | Gray Level Nonuniformity | 94.75 | 0.0002 |
|  |  | Run Length Nonuniformity | 123.18 | <0.0001 |
|  |  | Short Run High Gray Level Emphasis | 70.21 | 0.0195 |
|  | NGTDM | Busyness | 107.59 | 0.0011 |
|  |  | Coarseness | 184.29 | 0.0028 |
|  |  | Complexity | 69.59 | <0.0001 |
|  |  | Strength | 78.91 | 0.0161 |
|  | GLDM | Gray Level Nonuniformity | 39.42 | 0.0017 |
|  |  | Dependence Variance | 49.16 | 0.0035 |
|  |  | Small Dependence High Gray Level Emphasis | 60.99 | 0.0092 |

Abbreviations: eGFR, estimated glomerular filtration rate; GLCM, Gray-Level Co-occurrence Matrix; GLSZM, Gray Level Size Zone Matrix; GLRLM, Gray Level Run Length Matrix; NGTDM, Neighboring Gray Tone Difference Matrix; GLDM, Gray Level Dependence Matrix.

(b) The list of selected radiomic features, F-value, and p-value for eGFR20 model

| model | Feature class | Feature name | F-value | p-value |
| --- | --- | --- | --- | --- |
| eGFR20 | First Order | Minimum | 47.04 | <0.0001 |
|  |  | 10Percentile | 28.02 | <0.0001 |
|  |  | 90Percentile | 28.80 | <0.0001 |
|  |  | Mean | 31.91 | <0.0001 |
|  |  | Median | 25.57 | <0.0001 |
|  |  | Range | 20.27 | <0.0001 |
|  |  | RootMeanSquared | 30.71 | <0.0001 |
|  | Shape2D | Perimeter | 35.45 | <0.0001 |
|  |  | Perimeter Surface Ratio | 19.27 | <0.0001 |
|  |  | Sphericity | 29.61 | <0.0001 |
|  |  | Spherical Disproportion | 25.55 | <0.0001 |
|  |  | Maximum Diameter | 48.37 | <0.0001 |
|  |  | Major Axis Length | 48.02 | <0.0001 |
|  |  | Minor Axis Length | 20.12 | <0.0001 |
|  |  | Elongation | 21.97 | <0.0001 |
|  | GLSZM | Small Area Emphasis | 24.20 | <0.0001 |
|  |  | Gray Level Nonuniformity | 27.24 | <0.0001 |
|  |  | Size Zone Nonuniformity | 55.28 | <0.0001 |
|  |  | Size Zone Nonuniformity Normalized | 15.21 | 0.0001 |
|  |  | High Gray Level Zone Emphasis | 13.61 | 0.0002 |
|  |  | Small Area High Gray Level Emphasis | 39.40 | <0.0001 |
|  | GLRLM | Gray Level Nonuniformity | 19.30 | <0.0001 |
|  |  | Run Length Nonuniformity | 24.04 | <0.0001 |
|  |  | Short Run Low Gray Level Emphasis | 9.77 | 0.0018 |
|  | NGTDM | Busyness | 14.50 | 0.0002 |
|  |  | Coarseness | 10.25 | 0.0014 |
|  |  | Complexity | 25.17 | <0.0001 |
|  |  | Strength | 8.34 | 0.004 |
|  | GLDM | Gray Level Nonuniformity | 11.83 | 0.0006 |
|  |  | Dependence Nonuniformity | 8.01 | 0.0048 |
|  |  | Dependence Variance | 24.10 | <0.0001 |
|  |  | Small Dependence High Gray Level Emphasis | 11.91 | 0.0006 |

Abbreviations: eGFR, estimated glomerular filtration rate; GLCM, Gray-Level Co-occurrence Matrix; GLSZM, Gray Level Size Zone Matrix; GLRLM, Gray Level Run Length Matrix; NGTDM, Neighboring Gray Tone Difference Matrix; GLDM, Gray Level Dependence Matrix.

**Supplement 2.** The result of Image-based analysis.

**(a) *eGFR10 model***

***Image-based analysis***

Accuracy, sensitivity, specificity, PPV, NPV, F-score, AUC-ROC, logarithmic loss, and kappa score for the test dataset were 0.71 (95%CI; 0.69–0.74), 0.62 (0.58–0.66), 0.81 (0.77–0.83), 0.76 (0.72–0.79), 0.68 (0.64–0.71), 0.68 (0.64–0.73), 0.75 (0.70–0.80), 0.628 (0.624–0.632), and 0.43 (0.40–0.45), respectively. Here are the performance results of the CNN models, along with the outcomes of the McNemar and DeLong tests. When compared with the CNN models using tumor images, the vViT model showed statistically different contingency tables than VGG16 (p=0.011), ResNet50 (p=0.040), and DenseNet121 (p<0.0001). The AUC-ROC of the vViT model showed statistically higher results than VGG16 (p=0.003), ResNet50 (p<0.0001), and DenseNet121 (p<0.0001). When using tumor and kidney images, the vViT model showed statistically different contingency tables than VGG16 (p=0.005), ResNet50 (p<0.0001), and DenseNet121 (p=0.003) compared to the CNN models. The AUC-ROC of the vViT model was statistically higher than VGG16 (p=0.050) and ResNet50 (p=0.004). By the permutation feature importance, sectors were ranked in descending order as Personal health record (difference between the original accuracy and accuracy calculated using permutated dataset=0.048, 95%CI 0.046–0.050), Comorbidity (0.050, 0.048–0.051), Habit (-0.060, -0.062 to -0.058), Surgical (0.219, 0.218–0.221), Radiomic (0.227, 0.225–0.229), Tumor (-0.000, -0.002 to 0.002), and Kidney and tumor (-0.030, -0.032 to -0.028) sectors. Supplementary Figure 2a and 2b show ROC and the permutation feature importance of each sector for the test dataset, respectively. Supplementary Table 1a shows the statistics of each sector. The detailed results of the comparison with the CNN model are shown in Supplementary Table 2a.

**(b) *eGFR20 model***

***Image-based analysis***

Accuracy, sensitivity, specificity, PPV, NPV, F-score, AUC-ROC, logarithmic loss, and kappa score for the test dataset were 0.83 (95%CI; 0.80–0.85), 0.84 (0.79–0.86), 0.82 (0.77–0.85), 0.82 (0.78–0.85), 0.84 (0.79–0.86), 0.83 (0.78–0.88), 0.84 (0.78–0.89), 1.15 (1.14–1.15), and 0.66 (0.64–0.68), respectively. Here are the performance results of the CNN models, along with the outcomes of the McNemar and DeLong tests. When compared with the CNN models using tumor images, the vViT model showed contingency tables that were statistically different from VGG16 (p<0.0001) and ResNet50 (p=0.027). The AUC-ROC of the vViT model was statistically higher than VGG16 (p=0.001), ResNet50 (p=0.003), and DenseNet121 (p<0.0001). When using tumor and kidney images, the vViT model showed contingency tables that were statistically different from VGG16 (p<0.0001) and ResNet50 (p=0.001) compared to the CNN models. The AUC-ROC of the vViT model was statistically higher than VGG16 (p<0.0001), ResNet50 (p<0.0001), and DenseNet121 (p<0.0001). By the permutation feature importance, sectors were ranked in descending order as Personal health record (difference between the original accuracy and accuracy calculated using permutated dataset=0.001, 95%CI -0.001 to 0.003), Comorbidity (0.004, 0.001–0.006), Habit (-0.012, -0.014 to -0.010), Surgical (0.328, 0.326–0.330), Radiomic (0.312, 0.310–0.314), Tumor (-0.0004, -0.003 to 0.002), and Kidney and tumor (0.026, 0.024–0.028) sectors. Supplementary Figure 2c and 2d show ROC and the permutation feature importance of each sector for the test dataset, respectively. Supplementary Table 1b shows the statistics of each sector. The detailed results of the comparison with the CNN model are shown in Supplementary Table 2b.

**Supplementary Figure 2.** The number of patients and images excluded by exclusion criteria and random selection. (a) shows an overview of the eGFR10 model, and (b) shows an overview of the eGFR20 model.

Abbreviations: eGFR, estimated glomerular filtration rate.

(a)


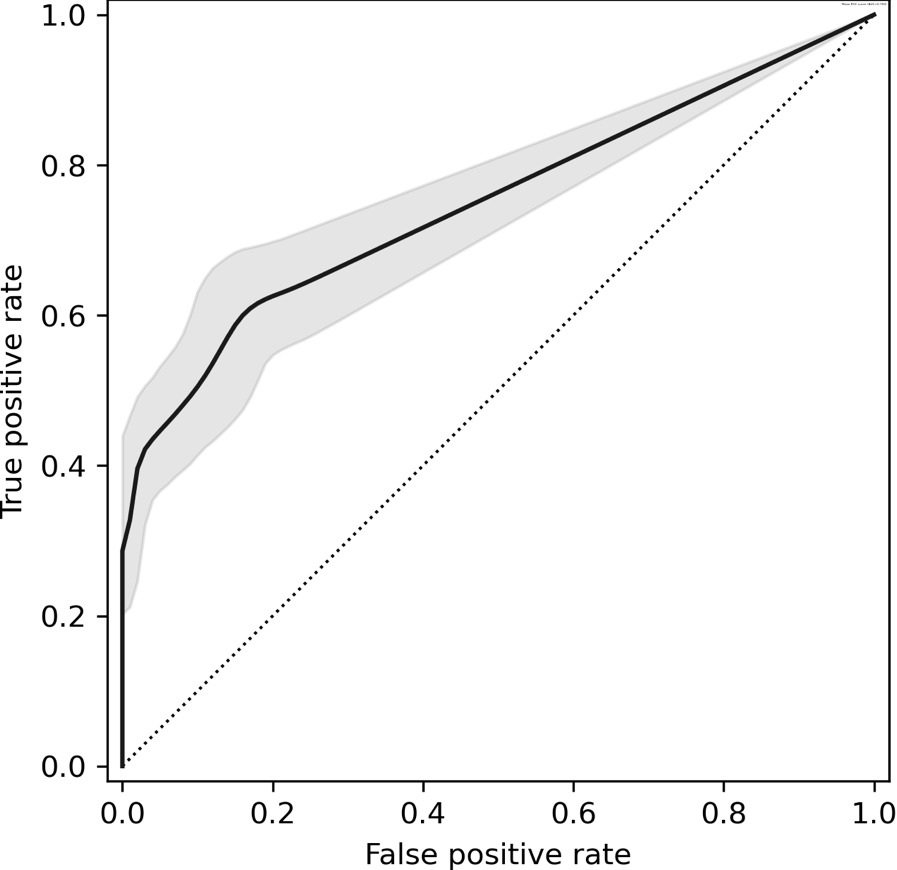


(b)


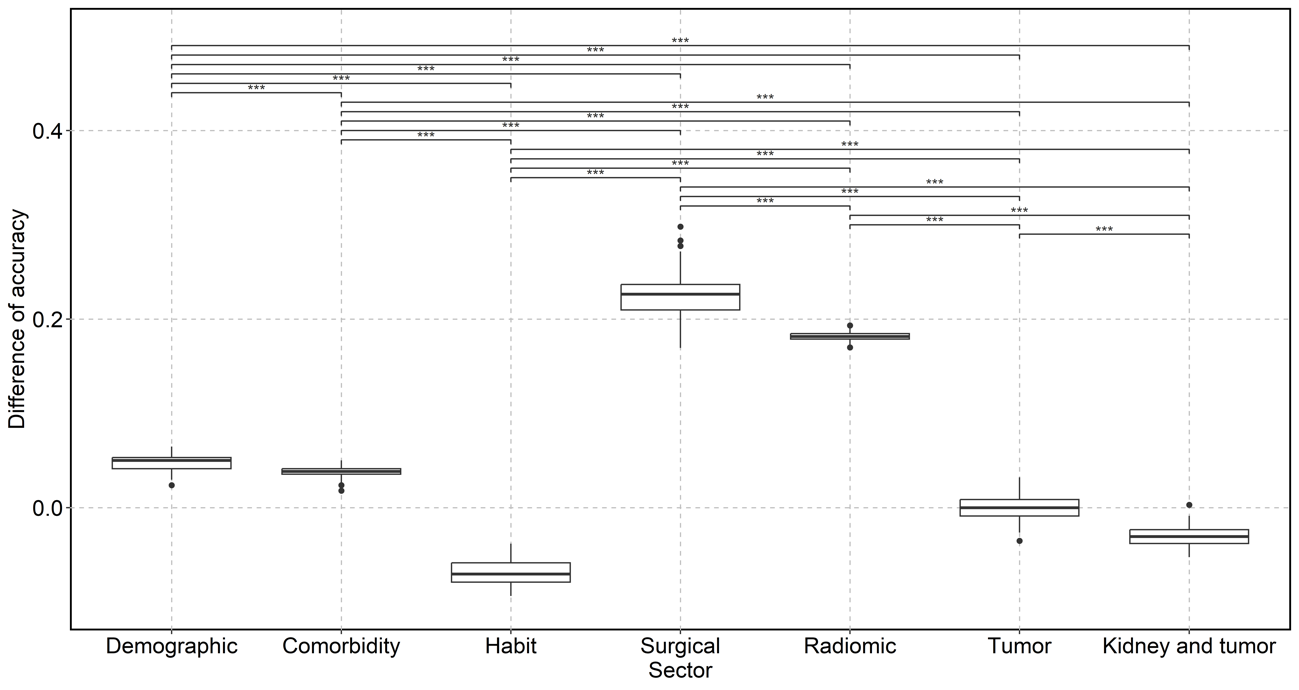


(c)


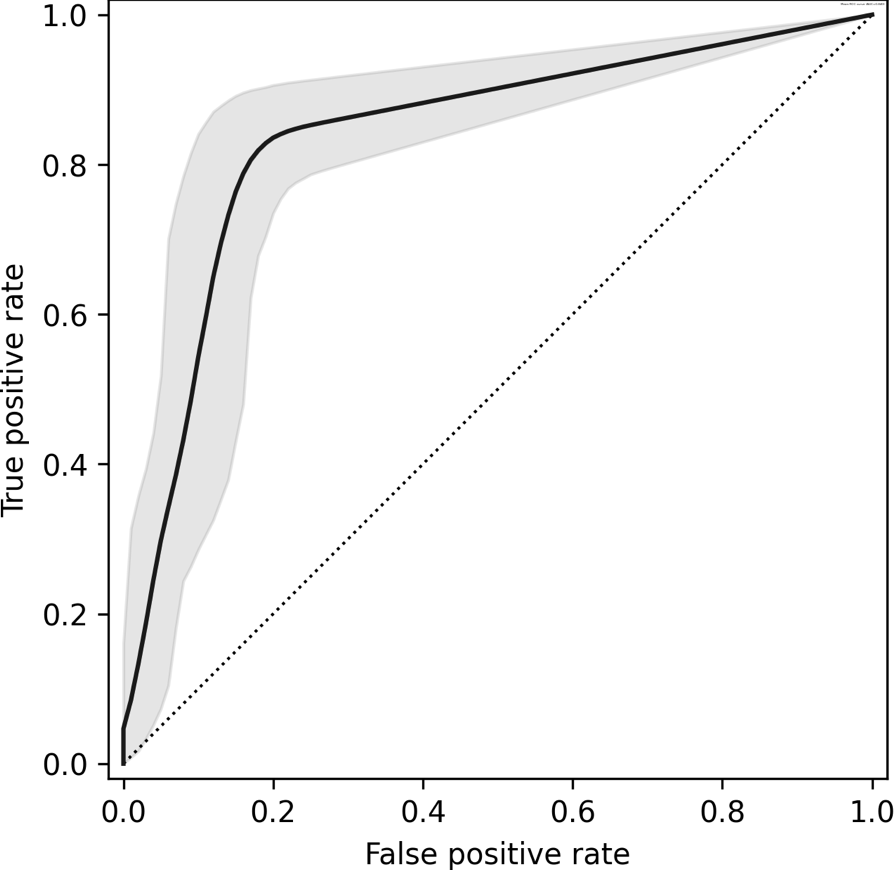


(d)


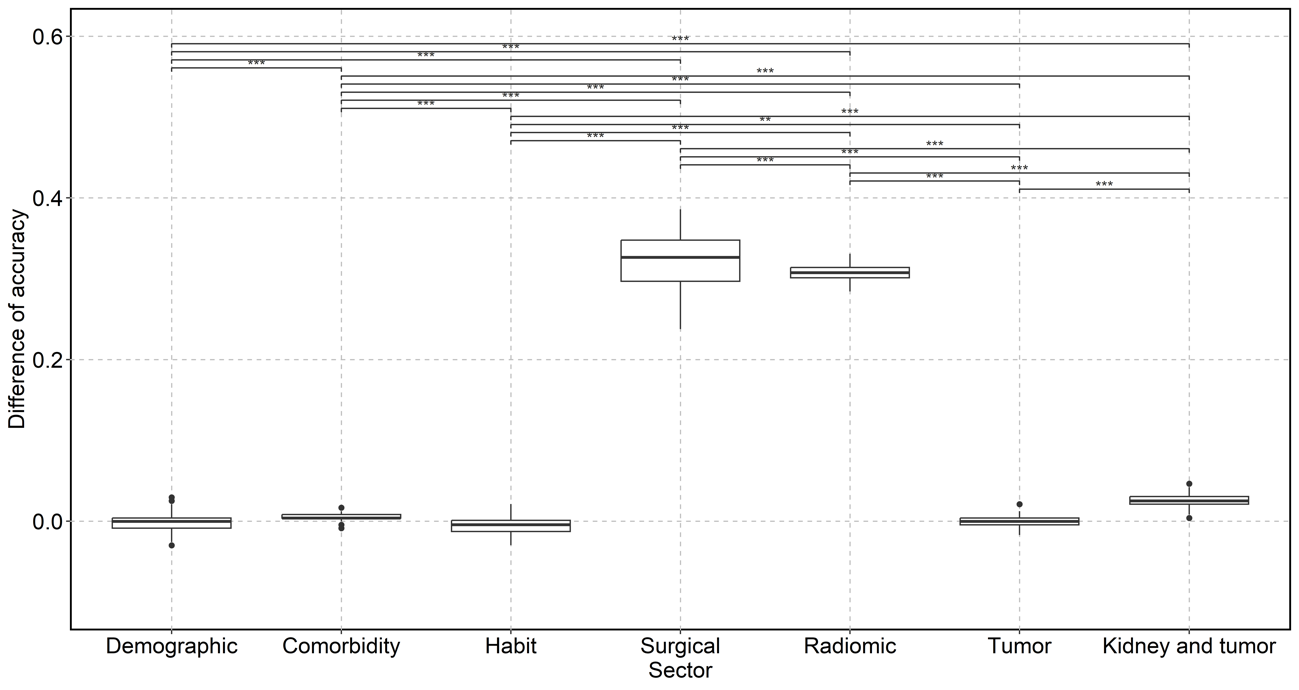


**Supplementary Table 2.** Statistical data for each sector

(a) Image-based analysis of predicting deterioration in postoperative renal function for the eGFR10 model

| Statistic  (95%CI) | Class token  sector | Demographic sector | Comorbidity sector | Habit sector | Surgical sector | Radiomic sector | Tumor sector | Kidney and tumor sector |
| --- | --- | --- | --- | --- | --- | --- | --- | --- |
| Accuracy | 0.629  (0.601–0.653) | 0.711  (0.684–0.733) | 0.673  (0.645–0.696) | 0.652  (0.625–0.676) | 0.725  (0.699–0.747) | 0.708  (0.681–0.730) | 0.652  (0.625–0.676) | 0.658  (0.631–0.682) |
| Sensitivity | 0.620  (0.580–0.654) | 0.620  (0.580–0.654) | 0.620  (0.580–0.654) | 0.620  (0.580–0.654) | 0.620  (0.580–0.654) | 0.620  (0.580–0.654) | 0.322  (0.290–0.362) | 0.433  (0.396–0.472) |
| Specificity | 0.637  (0.597–0.671) | 0.801  (0.763–0.826) | 0.725  (0.686–0.755) | 0.684  (0.644–0.716) | 0.830  (0.794–0.853) | 0.795  (0.757–0.820) | 0.982  (0.957–0.986) | 0.883  (0.849–0.901) |
| PPV | 0.631  (0.591–0.665) | 0.757  (0.713–0.787) | 0.693  (0.650–0.726) | 0.662  (0.621–0.696) | 0.785  (0.741–0.814) | 0.752  (0.708–0.782) | 0.948  (0.879–0.961) | 0.787  (0.732–0.820) |
| NPV | 0.626  (0.587–0.660) | 0.678  (0.642–0.708) | 0.656  (0.618–0.688) | 0.643  (0.604–0.676) | 0.686  (0.650–0.715) | 0.677  (0.640–0.706) | 0.592  (0.561–0.619) | 0.609  (0.576–0.638) |
| F-score | 0.625  (0.574–0.677) | 0.682  (0.632–0.731) | 0.654  (0.604–0.705) | 0.640  (0.590–0.691) | 0.693  (0.644–0.742) | 0.679  (0.630–0.729) | 0.480  (0.427–0.533) | 0.558  (0.506–0.611) |
| AUC-ROC | 0.717  (0.657–0.774) | 0.722  (0.664–0.781) | 0.720  (0.660–0.778) | 0.713  (0.654–0.771) | 0.729  (0.670–0.787) | 0.725  (0.665–0.783) | 0.726  (0.667–0.783) | 0.644  (0.579–0.708) |
| Logarithmic loss | 0.628  (0.624–0.633) | 0.622  (0.618–0.626) | 0.624  (0.620–0.628) | 0.628  (0.624–0.632) | 0.618  (0.614–0.622) | 0.622  (0.618–0.626) | 0.679  (0.675–0.683) | 0.681  (0.677–0.685) |
| Kappa score | 0.257  (0.232–0.283) | 0.421  (0.397–0.445) | 0.345  (0.320–0.370) | 0.304  (0.279–0.329) | 0.450  (0.427–0.474) | 0.415  (0.391–0.439) | 0.304  (0.279–0.329) | 0.316  (0.291–0.341) |

Abbreviations: 95% CI: 95% confidence interval; PPV: positive predictive value; NPV: negative predictive value; AUC-ROC: area under the curve of receiver-operating characteristics; eGFR, estimated glomerular filtration rate.

(b) Image-based analysis of predicting deterioration in postoperative renal function for the eGFR20 model

| Statistic  (95%CI) | Class token  sector | Demographic sector | Comorbidity sector | Habit sector | Surgical sector | Radiomic sector | Tumor sector | Kidney and tumor sector |
| --- | --- | --- | --- | --- | --- | --- | --- | --- |
| Accuracy | 0.831  (0.800–0.850) | 0.831  (0.800–0.850) | 0.826  (0.796–0.846) | 0.826  (0.796–0.846) | 0.822  (0.792–0.842) | 0.835  (0.805–0.854) | 0.835  (0.805–0.854) | 0.826  (0.796–0.846) |
| Sensitivity | 0.839  (0.793–0.864) | 0.839  (0.793–0.864) | 0.847  (0.801–0.872) | 0.847  (0.801–0.872) | 0.847  (0.801–0.872) | 0.839  (0.793–0.864) | 0.839  (0.793–0.864) | 0.814  (0.766–0.841) |
| Specificity | 0.822  (0.775–0.849) | 0.822  (0.775–0.849) | 0.805  (0.757–0.833) | 0.805  (0.757–0.833) | 0.797  (0.749–0.826) | 0.831  (0.784–0.856) | 0.831  (0.784–0.856) | 0.839  (0.793–0.864) |
| PPV | 0.825  (0.779–0.851) | 0.825  (0.779–0.851) | 0.813  (0.767–0.840) | 0.813  (0.767–0.840) | 0.806  (0.760–0.834) | 0.832  (0.785–0.858) | 0.832  (0.785–0.858) | 0.835  (0.787–0.861) |
| NPV | 0.836  (0.789–0.862) | 0.836  (0.789–0.862) | 0.841  (0.793–0.866) | 0.841  (0.793–0.866) | 0.839  (0.791–0.865) | 0.838  (0.791–0.863) | 0.838  (0.791–0.863) | 0.818  (0.772–0.845) |
| F-score | 0.832  (0.784–0.880) | 0.832  (0.784–0.880) | 0.830  (0.782–0.878) | 0.830  (0.782–0.878) | 0.826  (0.778–0.875) | 0.835  (0.788–0.883) | 0.835  (0.788–0.883) | 0.824  (0.775–0.873) |
| AUC-ROC | 0.839  (0.783–0.890) | 0.855  (0.802–0.904) | 0.851  (0.795–0.902) | 0.840  (0.782–0.893) | 0.832  (0.770–0.887) | 0.866  (0.813–0.913) | 0.860  (0.802–0.909) | 0.838  (0.778–0.891) |
| Logarithmic loss | 3.406  (3.400–3.412) | 1.423  (1.417–1.430) | 1.534  (1.528–1.541) | 1.525  (1.518–1.531) | 1.545  (1.538–1.551) | 1.456  (1.450–1.462) | 1.509  (1.503–1.515) | 0.906  (0.899–0.912) |
| Kappa score | 0.661  (0.637–0.685) | 0.661  (0.637–0.685) | 0.653  (0.628–0.677) | 0.653  (0.628–0.677) | 0.644  (0.620–0.668) | 0.669  (0.646–0.693) | 0.669  (0.646–0.693) | 0.653  (0.628–0.677) |

Abbreviations: 95% CI: 95% confidence interval; PPV: positive predictive value; NPV: negative predictive value; AUC-ROC: area under the curve of receiver-operating characteristics; eGFR, estimated glomerular filtration rate.

**Supplementary Table 3.** Comparison of performance between vViT and CNN models

(a) Comparison of image-based analysis with CNN models for predicting postoperative renal function decline in the eGFR10 model

| Statistic  (95%CI) | tumor | | | | kidney and tumor | | | |
| --- | --- | --- | --- | --- | --- | --- | --- | --- |
|  | vViT | VGG16 | ResNet50 | DenseNet121 | vViT | VGG16 | ResNet50 | DenseNet121 |
| Accuracy | 0.71  (0.69–0.74) | 0.640  (0.588-0.689) | 0.518  (0.465-0.570) | 0.523  (0.470-0.576) | 0.71  (0.69–0.74) | 0.626  (0.573-0.675) | 0.614  (0.561-0.664) | 0.617  (0.564-0.667) |
| Sensitivity | 0.62  (0.58–0.66) | 0.620  (0.567-0.670) | 0.345  (0.297-0.397) | 0.626  (0.573-0.675) | 0.62  (0.58–0.66) | 0.643  (0.591-0.692) | 0.807  (0.762-0.845) | 0.637  (0.585-0.687) |
| Specificity | 0.81  (0.77–0.83) | 0.661  (0.609-0.709) | 0.690  (0.639-0.737) | 0.421  (0.370-0.474) | 0.81  (0.77–0.83) | 0.608  (0.556-0.658) | 0.421  (0.370-0.474) | 0.596  (0.544-0.647) |
| PPV | 0.76  (0.72–0.79) | 0.646  (0.594-0.695) | 0.527  (0.474-0.579) | 0.519  (0.467-0.572) | 0.76  (0.72–0.79) | 0.621  (0.569-0.671) | 0.582  (0.529-0.633) | 0.612  (0.560-0.662) |
| NPV | 0.68  (0.64–0.71) | 0.635  (0.583-0.684) | 0.513  (0.460-0.566) | 0.529  (0.476-0.582) | 0.68  (0.64–0.71) | 0.630  (0.578-0.680) | 0.686  (0.635-0.733) | 0.622  (0.569-0.672) |
| F-score | 0.68  (0.64–0.73) | 0.633  (0.581-0.682) | 0.417  (0.366-0.470) | 0.568  (0.515-0.619) | 0.68  (0.64–0.73) | 0.632  (0.580-0.682) | 0.676  (0.625-0.724) | 0.625  (0.572-0.674) |
| p-value | - | 0.011 | 0.040 | <0.0001 | - | 0.005 | <0.0001 | 0.003 |
| AUC-ROC | 0.75  (0.70–0.80) | 0.666  (0.615-0.714) | 0.541  (0.488-0.593) | 0.551  (0.498-0.603) | 0.75  (0.70–0.80) | 0.675  (0.623-0.722) | 0.640  (0.588-0.689) | 0.690  (0.639-0.737) |
| p-value | - | 0.003 | <0.0001 | <0.0001 |  | 0.050 | 0.004 | 0.119 |

Abbreviations: CNN: convolutional neural network; eGFR, estimated glomerular filtration rate, 95%CI: 95% confidence interval; vVIT: variable Vision Transformer; VGG16: visual geometry group (VGG) 16; ResNet50: Residual Networks (ResNet)50; DenseNet121: Dense Convolutional Network (DenseNet)121; PPV: positive predictive value; NPV: negative predictive value; AUC-ROC: area under the curve of receiver-operating characteristics.

(b) Comparison of image-based analysis with CNN models for predicting postoperative renal function decline in the eGFR20 model

| Statistic  (95%CI) | tumor | | | | kidney and tumor | | | |
| --- | --- | --- | --- | --- | --- | --- | --- | --- |
|  | vViT | VGG16 | ResNet50 | DenseNet121 | vViT | VGG16 | ResNet50 | DenseNet121 |
| Accuracy | 0.83  (0.80–0.85) | 0.657  (0.594-0.714) | 0.695  (0.633-0.750) | 0.695  (0.633-0.750) | 0.83  (0.80–0.85) | 0.568  (0.504-0.629) | 0.661  (0.598-0.718) | 0.627  (0.564-0.686) |
| Sensitivity | 0.84  (0.79–0.86) | 0.432  (0.371-0.496) | 0.727  (0.667-0.780) | 0.702  (0.641-0.757) | 0.84  (0.79–0.86) | 0.305  (0.250-0.367) | 0.805  (0.750-0.851) | 0.695  (0.633-0.750) |
| Specificity | 0.82  (0.77–0.85) | 0.881  (0.834-0.917) | 0.788  (0.732-0.835) | 0.720  (0.660-0.774) | 0.82  (0.77–0.85) | 0.831  (0.777-0.873) | 0.517  (0.453-0.580) | 0.559  (0.496-0.621) |
| PPV | 0.82  (0.78–0.85) | 0.785  (0.728-0.832) | 0.602  (0.538-0.662) | 0.669  (0.607-0.726) | 0.82  (0.78–0.85) | 0.643  (0.580-0.701) | 0.625  (0.562-0.684) | 0.612  (0.548-0.672) |
| NPV | 0.84  (0.79–0.86) | 0.608  (0.545-0.668) | 0.664  (0.602-0.721) | 0.685  (0.624-0.741) | 0.84  (0.79–0.86) | 0.544  (0.481-0.607) | 0.726  (0.666-0.779) | 0.647  (0.584-0.705) |
| F-score | 0.83  (0.78–0.88) | 0.557  (0.494-0.619) | 0.740  (0.680-0.791) | 0.705  (0.644-0.760) | 0.83  (0.78–0.88) | 0.414  (0.353-0.478) | 0.704  (0.643-0.758) | 0.651  (0.588-0.709) |
| p-value | - | <0.0001 | 0.027 | 0.708 | - | <0.0001 | 0.001 | 0.130 |
| AUC-ROC | 0.84  (0.78–0.89) | 0.716  (0.655-0.770) | 0.721  (0.661-0.774) | 0.702  (0.641-0.757) | 0.84  (0.78–0.89) | 0.664  (0.601-0.721) | 0.696  (0.634-0.751) | 0.662  (0.599-0.719) |
| p-value | - | 0.001 | 0.003 | <0.0001 |  | <0.0001 | <0.0001 | <0.0001 |

Abbreviations: CNN: convolutional neural network; eGFR, estimated glomerular filtration rate, 95%CI: 95% confidence interval; vVIT: variable Vision Transformer; VGG16: visual geometry group (VGG) 16; ResNet50: Residual Networks (ResNet)50; DenseNet121: Dense Convolutional Network (DenseNet)121; PPV: positive predictive value; NPV: negative predictive value; AUC-ROC: area under the curve of receiver-operating characteristics.
